# Der OPTIMAL-Trial zur Tranexamsäure im herzchirurgischen OP – Hilft viel auch viel?

**DOI:** 10.1007/s00101-022-01216-6

**Published:** 2022-10-28

**Authors:** J. Grabert, M. Velten

**Affiliations:** Klinik für Anästhesiologie und Operative Intensivmedizin, Universitätsklinikum Bonn, Venusberg-Campus 1, 53127 Bonn, Deutschland

## Originalpublikation

Shi J, Zhou C, Pan W et al (2022) Effect of High- vs. Low-Dose Tranexamic Acid Infusion on Need for Red Blood Cell Transfusion and Adverse Events in Patients Undergoing Cardiac Surgery. JAMA 328(4):336–347

## Zusammenfassung der Studie

Die Gabe von Tranexamsäure (TXA) im Rahmen herzchirurgischer Operationen mit und ohne extrakorporale Zirkulation (EKZ) reduziert perioperative Blutungskomplikationen und dadurch bedingte Transfusionen [1, 2]. Derzeit existieren keine Empfehlungen in Bezug auf Dosierung und Applikationsart. Mit dem OPTIMAL-Trial werden eine hoch-dosierte mit einer niedrig-dosierten TXA-Gabe verglichen.

Die multizentrische, doppelt verblindete, randomisierte OPTIMAL-Studie (Outcome impact of different tranexamic acid regimens in cardiac surgery with cardiopulmonary bypass) vergleicht zwei Tranexamsäuredosierungskonzepte bei elektiven kardiochirurgischen Eingriffen mit EKZ. Von Dezember 2018 bis April 2021 wurden in 4 chinesischen Zentren 3079 erwachsene Patienten eingeschlossen. Die Hochdosistherapie mit TXA bestand aus einem Bolus nach Anästhesieinduktion (30 mg/kgKG), Erhaltungsdosis (16 mg/kgKG und h) und einem Bolus zum Beginn der EKZ (2 mg/kgKG). Die niedrig dosierte Therapie mit TXA bestand aus einem Bolus (10 mg/kgKG), einer Erhaltungsdosis (2 mg/kgKG und h) und einem Bolus zu Beginn der EKZ (1 mg/kgKG).

Primärer Endpunkt hinsichtlich Effektivität war die Transfusionsrate von Erythrozytenkonzentraten nach 30 Tagen; der primäre kombinierte Endpunkt hinsichtlich Sicherheit beinhaltete Krampfanfälle, Nierenfunktionsstörung, thrombembolische Ereignisse und Mortalität. Sekundäre Endpunkte beinhalteten Transfusionsvolumen, Blutungskomplikationen, operative Revisionen, Beatmungsdauer sowie Krankenhaus- und intensivstationäre Behandlungsdauer.

Die Kohorten waren hinsichtlich Vorerkrankungen, Vortherapie und Operationsart vergleichbar. Die Anzahl transfundierter Erythrozytenkonzentrate (21,8 % vs. 26,0 %, *p* = 0,004) und das transfundierte Volumen (Median 0 ml vs. 0 ml, *p* = 0,01) waren in der Hochdosisgruppe signifikant niedriger. Bezogen auf den Komposit-Endpunkt Sicherheit traten in der Hochdosisgruppe kumulativ signifikant mehr Komplikationen auf (17,6 % vs. 16,8 %, *p* = 0,003). In der Einzelanalyse war statistisch lediglich für Krampfanfälle eine signifikante Häufung in der Hochdosisgruppe (1,0 % vs. 0,4 %, *p* = 0,05) zu detektieren. Beide Gruppen unterschieden sich in den sekundären Endpunkten nicht.

Weitere Untersuchungen nach 6 und 12 Monaten sind geplant. Die Autoren konkludieren, dass die Hochdosistherapie mit TXA bezüglich des Sicherheitsprofils nicht unterlegen ist und bezüglich der Effektivität zu einer moderaten Reduktion an Transfusionen führt.

## Ihr Kommentar zur Studie

Der Einsatz von Tranexamsäure hat in den letzten Jahren in weiten Bereichen an Bedeutung gewonnen und wird u. a. in den Leitlinien Polytrauma und postpartale Hämorrhagie empfohlen [3–5]. In neueren Studien wird der standardisierte Einsatz bei nicht herzchirurgischen Patienten kritisch hinterfragt und ein individualisiertes Vorgehen, das mögliche Vorteile gegen thrombembolische Komplikationen abwägt, empfohlen [6]. Trotzdem ist die perioperative Gabe bei herzchirurgischen Eingriffen etabliert, die aktuellen Leitlinien beinhalten jedoch keine Dosierungsempfehlungen [1, 2]. Diesem Umstand widmen sich Shi et al. in der vorliegenden OPTIMAL-Studie.

Die beiden untersuchten TXA-Dosierungen wurden konsequent umgesetzt, sodass in den Gruppen tatsächlich signifikant unterschiedliche Mengen appliziert wurden. Die Anzahl transfundierter Erythrozytenkonzentrate (EK) wird in der Hochdosisgruppe als signifikant niedriger angegeben (*p* = 0,004). Das tatsächlich transfundierte EK-Volumen beträgt im Median in beiden Gruppen 0 ml. Betrachtet man die Verteilung im Boxplot des Supplements, bewegt sich die Masse der Punkte in beiden Gruppen zwischen 50 ml und 200 ml EK, sodass sich die Frage nach der klinischen Relevanz in der Transfusion eines EK stellt.

Die intraoperative Gabe von Gerinnungsprodukten, postoperative Drainageverluste und Häufigkeit von Revisionsoperationen war in beiden Gruppen vergleichbar.

Die Autoren postulieren eine Noninferiorität der Hochdosisgruppe in Bezug auf die Sicherheit, obwohl in der Subgruppenanalyse das vermehrte Auftreten von Krampfanfällen (*p* = 0,05) berichtet wird. Soweit aus dem Supplement hervorgeht, hat kein Patient ein bleibendes neurologisches Defizit, jedoch muss kritisch hinterfragt werden, ob die hochdosierte Gabe von TXA, die auf Grundlage dieser Studie nur wenige EK-Transfusion einspart, die Inkaufnahme einer höheren Inzidenz von Krampfanfällen rechtfertigt.

Die Patienten in beiden Gruppen sind vergleichbar. Insgesamt fällt jedoch ein junges Alter (im Mittel 53 Jahre) bei einem BMI von im Mittel 24,4 kg/m^2^ auf. In beiden Gruppen sind die Hälfte der Patienten NYHA-Klasse 2, während eine ASA-Klassifizierung nicht angegeben ist. Damit sind die Patienten auffällig jung, schlank und scheinbar wenig beeinträchtigt, während der durchschnittliche herzchirurgische Patient in Deutschland älter, korpulenter und weniger belastbar ist [7–9]. Es ist Spekulation, ob ältere Patienten mit einem vermutlich niedrigeren Hb von der Hochdosistherapie mit TXA profitieren könnten, oder sich bei wahrscheinlich schon vorbestehenden Organschäden die TXA-Nebenwirkungen deutlicher ausprägen würden.

Kritisch anzumerken ist, dass die Prozentangaben in den Tabellen 1 und 2 rechnerisch nicht immer nachzuvollziehen sind und somit nicht immer klar ist, auf welchen Teil sich die Prozentangabe bezieht.

## Fazit für die Praxis

Zusammenfassend ist der OPTIMAL-Trial eine groß angelegte und für die Frage nach der optimalen TXA-Dosierung gut angelegte Studie, die schlussendlich aber kein überzeugendes Ergebnis liefern kann. Die Reduktionen sowohl der EK-Anzahl als auch des transfundierten Volumens sind gering, während signifikant mehr Krampfanfälle auftraten. Zudem ist spekulativ, wie sich diese Daten auf eine in der Regel ältere, korpulentere und vermutlich kränkere Klientel übertragen lassen.
